# Quantitative study of H protein lipoylation of the glycine cleavage system and a strategy to increase its activity by co-expression of LplA

**DOI:** 10.1186/s13036-019-0164-5

**Published:** 2019-04-23

**Authors:** Xinyi Zhang, Mei Li, Yingying Xu, Jie Ren, An-Ping Zeng

**Affiliations:** 1 Beijing Advanced Innovation Center for Soft Matter Science and Engineering, Beijing University of Chemical Technology, North Third Ring Road 15, Beijing, 100029 China; 2 Institute of Bioprocess and Biosystems Engineering, Hamburg University of Technology, Denickestrasse 15, D-21073 Hamburg, Germany

**Keywords:** Glycine cleavage system, H protein, Lipoylation, LplA, Formate

## Abstract

Glycine cleavage system (GCS) plays a key role in one-carbon (C1) metabolism related to the biosynthesis of a number of key intermediates with significance in both biomedicine and biotechnology. Despite extensive studies of the proteins (H, T, P and L) involved and the reaction mechanisms of this important enzyme complex little quantitative data are available. In this work, we have developed a simple HPLC method for direct analysis and quantification of the apo- and lipoylated forms (H_apo_ and H_lip_) of the shuttle protein H, the latter (H_lip_) is essential for the function of H protein and determines the activity of GCS. Effects of temperature, concentrations of lipoic acid and H_apo_ and the expression of H protein on its lipoylation were studied. It is found that H_lip_ is as low as only 20–30% of the total H protein with lipoic acid concentration in the range of 10–20 μM and at a favorable temperature of 30 °C. Furthermore, H_apo_ seems to inhibit the overall activity of GCS. We proposed a strategy of co-expressing LplA to improve the lipoylation of H protein and GCS activity. With this strategy the fraction of H_lip_ was increased, for example, from 30 to 90% at a lipoic acid concentration of 20 μM and GCS activity was increased by more than 2.5 fold. This work lays a quantitative foundation for better understanding and reengineering the GCS system.

## Introduction

**Fig. 1:**
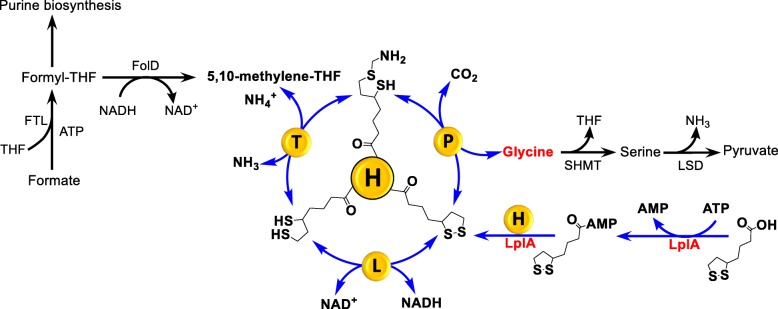
Glycine cleavage system (GCS) with H protein as a shuttle among its components, also shown are the lipoylation of H protein and the roles of GCS in the utilization of formate and purine biosynthesis

Glycine cleavage system (GCS) is the major degradation pathway of glycine widely distributed in animals, plants and bacteria (Kikuchi et al. 2008). In GCS glycine is enzymatically cleaved into CO_2_, NH_4_^+^, and a methylene group (Fig. 1). The methylene group is accepted by tetrahydrofolate (THF), forming 5,10-methylene-THF as the one-carbon (C1) source for purine synthesis and cell growth, and yielding one molecule of NADH as reducing power [1]. GCS also catalyzes the reversible reaction of glycine synthesis from CO_2_, ammonium, 5,10-methylene-THF and NADH, especially in anaerobic bacteria such as *Clostridium acidiurici* [2, 3].

Bar-Even et al. (2013) proposed the use of reversed GCS reactions as a central part of the so-called reductive glycine pathway as the most promising pathway for developing a synthetic formatotropic microorganism for the use of formate and CO_2_ [4]. Recently, the reversed GCS reactions have been successfully used to construct novel C1 assimilation pathways in *Escherichia coli* for the use of formate and CO_2_ [5–11]. To this end, endogenous GCS and exogenous formyl-methenyl-methylenetetrahydrofolate synthetase were overexpressed in engineered *E. coli* to convert formate into glycine and serine, and then channeled into the central metabolism pathway. However, the reaction rate or flux of glycerin synthesis is still quite low and only about 10% of the carbon for cell growth can be supplied by the synthetic pathway. It is essential to better understand and reengineer GCS for a truly formatotrophic growth in both C1 utilization and CO_2_ fixation.

GCS consists of four enzymes: glycine decarboxylase (P protein), aminomethyl-transferase (T protein), dihydrolipoyl dehydrogenase (L protein) and a carrier protein (H protein) (Fig. 1) [12–14]. The H protein plays a pivotal role and interacts with the other three proteins through a lipoic acid arm bound to a lysine residue [15]. The lipoyl group is the “true” shuttle which carries the aminomethyl group between the P protein and the T protein, and regenerates through the L protein yielding NADH at the same time. It may therefore play a key role in determine the overall reaction rate. Two mechanisms are known to perform lipoylation reaction in nature: one is to transfer the lipoyl group from lipoylated E2 protein of keto-acid dehydrogenase catalyzed by lipoyl (octanoyl) transferase (EC 2.3.1.181LipB) [16], and the other is lipoylation with exogenous lipoic acid under the involvement of ATP and lipoate-protein ligase A (EC 6.3.1.20, LplA) [17]. Fujiwara and Motokawa (1990) developed a method to quantify the rate of H protein lipoylation via mapping digestion peptides of the apo-form of H protein (H_apo_) and the lipolated H protein (H_lip_) using HPLC and mass spectroscopy [18]. They proved that only a trace amount of the H protein was lipoylated when H protein was overexpressed in *E. coli* cultured without addition of lipoic acid. When the cells were cultured in medium supplemented with 30 μM lipoic acid, about 10% of the recombinant protein expressed had the correctly lipoylated active form, the other 10% were in an inactive aberrantly modified form, presumably with an octanoyl group [19], and the remaining 80% were the apo-form. However, Macherel *et. al*. (2010) reached different results: with the same expression vector (PET system) they obtained more than 90% of a recombinant pea H protein in the lipoylated form with 100 μM lipoic acid added. [20] They assumed that the difference might be due to the different induction time.

In engineered *E. coli* overexpressing GCS, the lipoylation rate of H protein is an important factor that may limit the C1 assimilation pathway. Despite intensive studies of GCS in the past, quantitative data and information are still scare regarding the interactions of the GCS components and potential limiting steps in both the forward and reversed reaction directions of GCS. In particular, uncertainties exist in literature regarding a potential inhibiting role of H_apo_ and the extent of H protein lipoylation under different conditions. In this work, we have developed a direct HPLC method for the analysis and quantification of H_apo_ and H_lip_ proteins and systematically examined the lipoylation of H protein and the corresponding GCS activity regarding the effects of H_apo_, H protein expression (inducer concentration, induction temperature and time), and lipoic acid concentration. Furthermore, we propose a strategy to enhance the lipoylation rate of H protein and the activity of GCS via co-expression of LplA.

## Material and methods

### Materials

NaCl, glycine, Tris, and HCl were analytical grade and purchased from Sinopharm Chemical Regent Co. LTD (Beijing, China). THF, NAD+, pyridoxal 5-phosphate monohydrate (PLP) were purchased from Sigma-Aldrich (Shanghai, China). Bicinchoninic acid (BCA) Protein Assay Kit was purchased from Beijing Solarbio Science & Technology Co. LTD (Beijing, China). Acetonitrile and trifluoroacetic acid (TFA) were chromatographic grade and purchased from J&K Scientific Ltd. (Beijing, China). Chemically competent cells of *E. coli* TOP10 and *E. coli* BL21(DE3) were purchased from Weidishengwu Ltd. (Beijing, China). In-fusion cloning was used for the ligation of sequence fragments to vector with the In-fusion HD Cloning Kit (Clontech Laboratories, Inc., US). Luria–Bertani (LB) liquid medium (tryptone 10 g/L, yeast extract 5 g/L and NaCl 10 g/L) and solid medium (1.5% agar) with kanamycin (100 μg/mL), ampicillin (100 μg/mL) were used for transformation, screening and cell growth.

### Plasmid construction

The gene encoding H protein was amplified from *E. coli* MG1655 cells by PCR with His-tag and constructed into the pET28a^+^ vector and pETduet-1 vector MCSI site by In-fusion cloning, yielding the plasmids pET28-H and pETduet-H, respectively. The gene encoding LplA was amplified from *E. coli* MG1655 cells by PCR and constructed into the pET28a^+^ vector and pETduet-H plasmid MCSII site by In-fusion cloning, yielding the plasmids pET28-LplA and pETduet-H-LplA, respectively. The plasmids were transferred into competent cells of *E. coli* BL21(DE3) for protein expression. Sequences for the primers and genes encoding for the H protein and LplA are given in Table 1.

**Table 1:** Sequences and primers for the cloning and expression of genes for the proteins pet28a-H_apo_, pet28a-T, pet28a-P, pet28a-L, pet28a-LplA, and pETduet-H-LplA

Primer	Oligonucleotide Sequence	Restriction Site	NCBI NO.
ecH-fwd	CATGCCATGGGCAGCAACGTACCAGCAGAACTGAAATAC	NcoI	WP_001295377.1
ecH-rev	CCGCTCGAGCTCGTCTTCTAACAATGCTTCGTATGC	XhoI	
ecT-fwd	CATGCCATGGCACAACAGACTCCTTTGTACGAACAA	NcoI	WP_099356926.1
ecT-rev	CCGCTCGAGCGCGACGGCTTTGCCGTTACGCACAAAAAC	XhoI	
ecP-fwd	CATGCCATGGGCACACAGACGTTAAGCCAGCTTGAAAAC	NcoI	WP_112929453.1
ecP-rev	CCGCTCGAGCTGGTATTCGCTAATCGGTACGCAGGAGCAG	XhoI	
ecL-fwd	CCGCTCGAGTTACTTCTTCTTCGCTTTCGGGTTC	XhoI	WP_110826218.1
ecL-rev	GGGAATTCCATATGATGAGTACTGAAATCAAAACTCAGGTCG	NdeI	
ecLplA-fwd	CCATGGGCTCCACATTACGCCTGCTCATCTCT	NcoI	WP_000105885.1
ecLplA-rev	CTCGAGCTACCTTACAGCCCCCGCCAT	XhoI	
pETduet-ecH-LplA			
H-fwd	CATGCCATGGGCAGCAACGTACC	NcoI	
H-rev	CCCAAGCTTGGCTTTGTTAGCAGCCGGATC	HindIII	
vector-fwd	GGCCACGCGATCGCTG	Infusion	
vector-rev	TATCCAATTGAGATCTGCCATATGTATATCTCCTTCTTAT		
Fragement-fwd	GATCTCAATTGGATAATGGGCTCCACATTACGCC		
Fragement-rev	AGCGATCGCGTGGCCCTACCTTACAGCCCCCGC		

### Expression of recombinant H-LplA protein

Cells harboring the plasmids pET28-H, pETduet-H, pET28-LplA and pETduet-H-LplA were grown at 37 °C in LB medium containing suitable antibiotics, in the presence of 0–100 μM lipoic acid, respectively. Induction of the target protein was started by adding 0.1–0.5 mM isopropyl β-D-1-thiogalactopyranoside (IPTG) when the optical density at 600 nm reached 0.6. The culture was then allowed to grow for additional 12 h at 18 °C or 30 °C, respectively. After medium removal by centrifugation at 10,000 x g for 5 min at 4 °C, the bacterial pellet was re-suspended in a phosphate buffer (100 mM, pH 7.0) and lysed by a Xinzhi JY92-IIN Ultrasonic Homogenizer. The supernatant was collected by centrifugation at 10,000 x g for 5 min at 4 °C and examined using SDS page. The H_apo_ protein and H_lip_ protein were purified using nucleophilic chromatography with a nickel column. The column was pre-equilibrated with lysis buffer (50 mM Tris, 10 mM imidazole, 300 mM NaCl, pH 7.5). The sample (30 mL) was loaded with a flow rate of 1.0 mL/min. After equilibration lysis buffer and wash buffer (50 mM Tris, 30 mM imidazole, 300 mM NaCl) were used to elute miscellaneous proteins, and then an elution buffer (50 mM Tris, 300 mM imidazole, 300 mM NaCl, pH 7.5) was used to obtain purified H_apo_ and H_lip_ proteins. Protein concentration was measured using the Bradford method [21].

### Expression of recombinant H, P, T and L proteins

Recombinant cells were incubated at 37 °C in Luria-Bertani medium containing 50 μg/mL kanamycin, until the OD_***600***_ reached about 0.7. The expression of recombinant H, P, T and L proteins followed the expression method described above for recombinant H-LplA protein with slight modification (Table 1). Sequences for genes encoding for the P, T and L proteins are given in Table 1. The culture for recombinant P, T and L proteins didn’t contain lipoic acid. Induction of the target protein was started by addition of 0.2 mM IPTG when the OD_***600***_ reached 0.7, the recombinant cells were then incubated for another 12 h at 30 °C.

### Enzyme purification

Cells were harvested by centrifugation, resuspended in lysis buffer [10 mM imidazole, 0.3 M NaCl, and 50 mM Tris-HCl (pH 7.8)], and lysed by sonication. The lysate was cleared by centrifugation, and the protein was purified using a column of chelating Sepharose Fast Flow (GE Healthcare Bio- Sciences Corp.) charged with Ni^2+^ ion. Cell lysate was applied to the column in lysis buffer, washed with buffer containing 30 mM imidazole, 0.3 M NaCl, and 50 mM Tris-HCl (pH 7.8), and eluted with 300 mM imidazole, 0.3 M NaCl, and 50 mM Tris-HCl (pH 7.8). Fractions containing the protein were pooled and dialyzed against Tris-HCl (100 mM, pH 7.0) and the protein fractions were collected and stored at − 80 °C. Protein concentration was measured using the BCA Protein Quantitation Kit.

Recombinant H-LplA protein (HL protein), and the proteins H, P, T and L protein were similarly expressed in *E. coli* strain BL21(DE3) and purified as described above.

### Separation and quantification of H_apo_ and H_lip_ proteins using HPLC

**Fig. 2:**
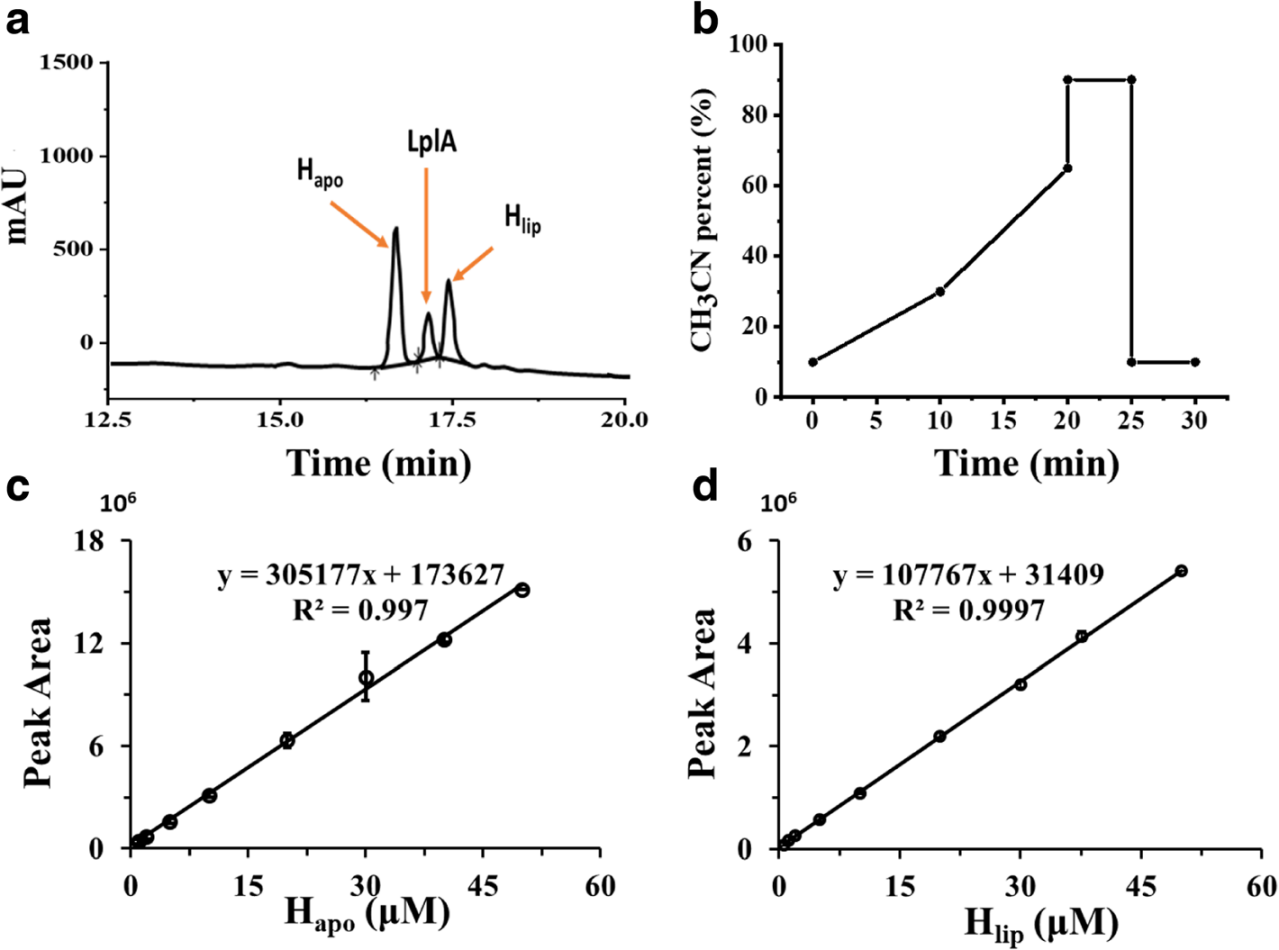
(**a**) Separation of H_apo_, H_lip_ and LplA proteins on HPLC; (**b**) The HPLC elution grogram; (**c**) Calibration curve for H_apo_ protein; (**d**) Calibration curve for H_lip_ protein

H_apo_ and H_lip_ proteins were separated using a Shimadzu LC-2030C system with a Shim-pack Inertsil WP300 C18 column (5 μm, 4.6 × 150 mm) and a 210 nm UV detector at 30 °C (Fig. 2a). The mobile phase was a mixture of acetonitrile and 0.1% trifluoroacetic acid solution with a flow rate of 1.0 mL/min. The ratio of the mobile phase with time is shown in Fig. 2b. BCA Protein Quantitation Kit was used to quantify the concentrations of H_apo_ and H_lip_ and to establish the calibration curves for using HPLC to measure these proteins. A good linear relationship between the peak area and the protein concentration was obtained for H_apo_ and H_lip_, respectively (Fig. 2c and d). Thus, HPLC can be used for a quantitative measurement of these proteins by using external standard method. The same method was also used to measure LplA (Fig. 2a).

### Determination of the glycine cleavage system

The rate of glycine cleavage was coupled to the NADH formation rate and it can be measured at 340 nm using a microplate reader. Briefly, the reaction mixture (200 uL) contained 1 mM NAD^+^, 1 mM THF, 0.1 mM PLP, 5.8 μM P protein, 13.4 μM T protein, 7.7 μM L protein, Tris-HCl (100 mM, pH 7.0) and different concentrations of H protein and HL protein (from 0.6 μM to 41.3 μM). The components were premixed and centrifuged before the reaction started. Then, 1 mM glycine was added to initiate the reaction. One enzyme activity unit (U) is defined as the amount of enzyme that produces 1 μmol of NADH per min.

### Determination of the influence of H_apo_ protein on the glycine cleavage system

H_apo_ protein was also used to probe the rate of the glycine cleavage system. H_apo_ protein was incubated without lipoic acid. The purification of H_apo_ protein followed the purification methods of other proteins. The reaction mixture (200 uL) contained 1 mM glycine, 1 mM NAD^+^, 1 mM THF, 0.1 mM PLP, 5.8 μM P protein, 13.4 μM T protein, 7.7 μM L protein, Tris-HCl (100 mM, pH 7.0), 6 μM HL protein and different concentrations of H_apo_ protein (from 0.6 μM to 6 μM). The components were premixed and centrifuged before the reactions started. And then, glycine was added to initiate the reaction.

## Results and discussion

### Effect of H_apo_ protein on GCS activity

**Fig. 3:**
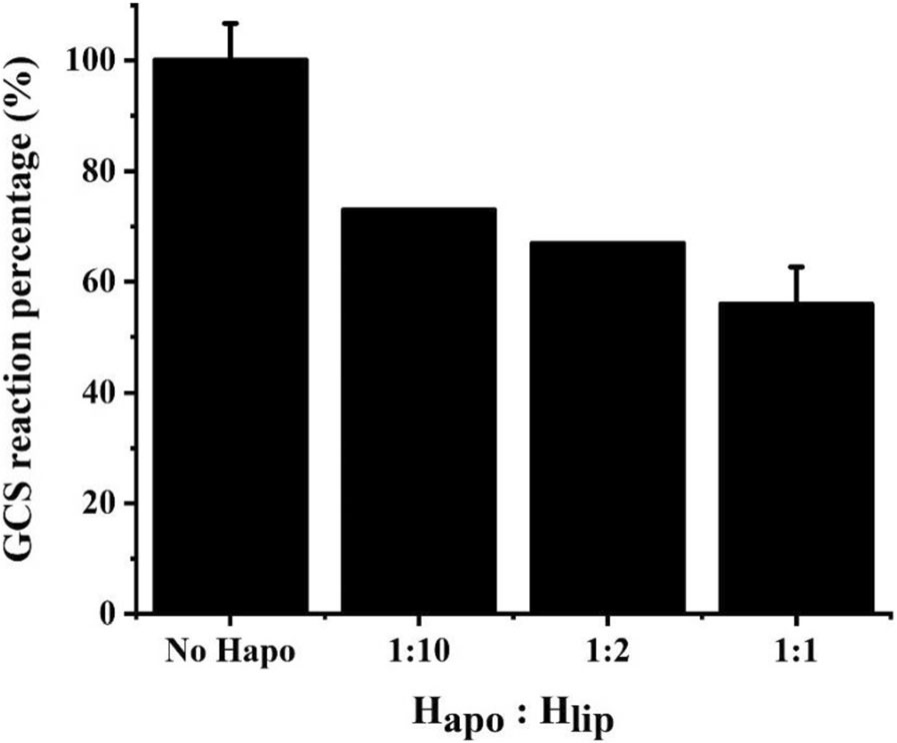
Effect of H_apo_:H_lip_ on the glycine cleavage activity of GCS

In the GCS system, H protein can only function after lipoylation. In the literature it was mentioned that the percent of H_apo_ in the total H protein affects the activity of GCS, but no experimental data were reported. [22] We determined the effect of different concentrations of H_apo_ protein on the degradation activity of glycine. It was found that when H_apo_: H_lip_ = 1:10, the GCS activity decreased by 25% (Fig. 3a.). When H_apo_: H_lip_ = 1:1, the GCS activity was reduced by 40%. This may be due to protein interaction among the four components of GCS: H_apo_ without the lipoic arm can also bind with other proteins, such as T protein, thereby acting as a competitive inhibitor for H_lip_. Whatever the exact reason(s) could be, it is clear that the lipoylation of H protein has a significant impact on the GCS system and needs more detailed study.

### Effects of expression conditions and lipoic acid concentration on the lipoylation of H protein

**Fig. 4:**
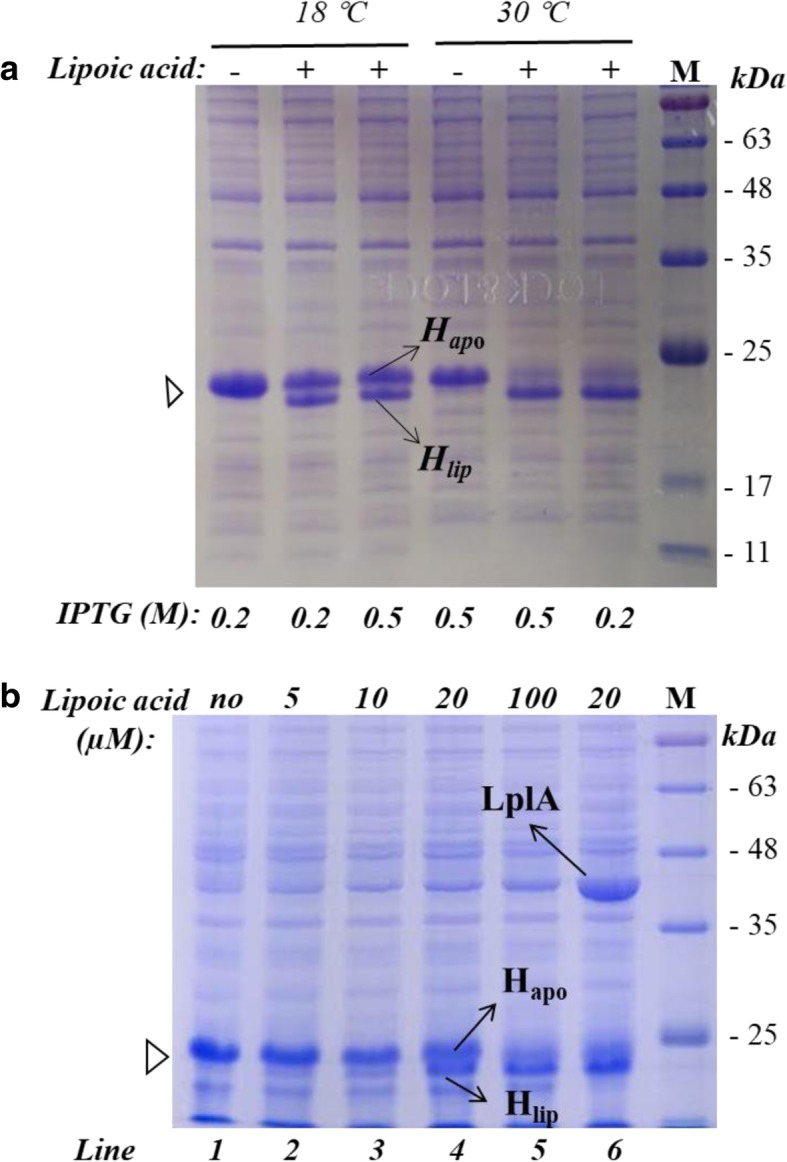
(**a**) Effects of temperature and concentration of the inducer IPTG on the lipoylation of H protein expressed in vivo; (**b**) Effect of lipoic acid concentration added into the culture on the lipoylation of H protein (lane 1–5, H protein was expressed in pET28a; lane 6, H protein and LplA was co-expressed in pETduet; the lipoic acid concentration added in this case was 20 μM)

As pointed out in the introduction, the rate of H protein in vivo lipoylation reported in literature was controversy. To clarify this point, we first checked the effects of temperature and the inducer IPTG concentration on the lipoylation of expressed H protein. The results are shown at Fig. 4. H_apo_ and H_lip_ proteins can be separated on SDS page with carefully controlled agar concentration at 12%. H_lip_ protein had a higher migration speed than H_apo_ protein on SDS page. When no lipoic acid was added into the culture, nearly no H_lip_ protein could be found on the SDS page at both 18 °C and 30 °C. With the addition of 100 μM lipoic acid into the culture, a small part of the H protein was lipoylated at 18 °C. The increase of IPTG from 0.2 mM to 0.5 mM didn’t significantly change the lipoylation under these conditions. In contrast, the majority of the H protein was lipoylated at 30 °C, with H_apo_ being at a very low level. At 30 °C the increase of IPTG concentration seemed also to have no effect on the lipoylation.

The concentration of lipoic acid added into the culture also has a significant effect on the lipoylation of H protein (Fig. 4b). It can be seen that with the increase of lipoic acid concentration, the lipoylation ratio of H protein was significantly increased. When the concentration of lipoic acid is lower than 10 μM, only a small amount of H protein was lipoylated. A part of the H protein was lipoylated when the lipoic acid concentration was increased to 20 μM. When the concentration reached 100 μM, most of the H protein was lipoylated. These results indicated that intracellular and free lipoic acid may not be sufficient to lipoylate the H protein synthesized under certain conditions. Another possible reason would be the limited activity of the protein LplA (Fig. 1). The results presented above are qualitative. For better understanding the regulation of GCS, especially for reengineering it for synthetic metabolic pathways for C1 carbon uses, it is desired to have more quantitative data and knowledge.

### Quantitative assessment of H protein lipoylation and activity of LplA

In literature, H_apo_ and H_lip_ proteins were only analyzed using mass spectrometry and their activity were indirectly measured with P protein [19]. The methods are cumbersome and can only be applied to purified proteins. We have developed a relatively simple method for quantitative measurement of H_apo_ and H_lip_ proteins using HPLC (Fig. 2). Based on the difference in the hydrophobicity of the lipoic acid side chain, a WP300 C18 column with reversed-phase macropores was selected to separate H_apo_ and H_lip_ with proper gradients of acetonitrile and water. It was found that the two proteins have a good resolution with a gradient solution containing acetonitrile in the range of 45–55%. The conditions were further optimized so that the LplA protein can also be separated, as shown in Fig. 2a. In addition, the method can analyze crude enzyme solution of H_apo_ and H_lip_ proteins without purification, which avoids the problem of protein loss during the purification process.

**Fig. 5:**
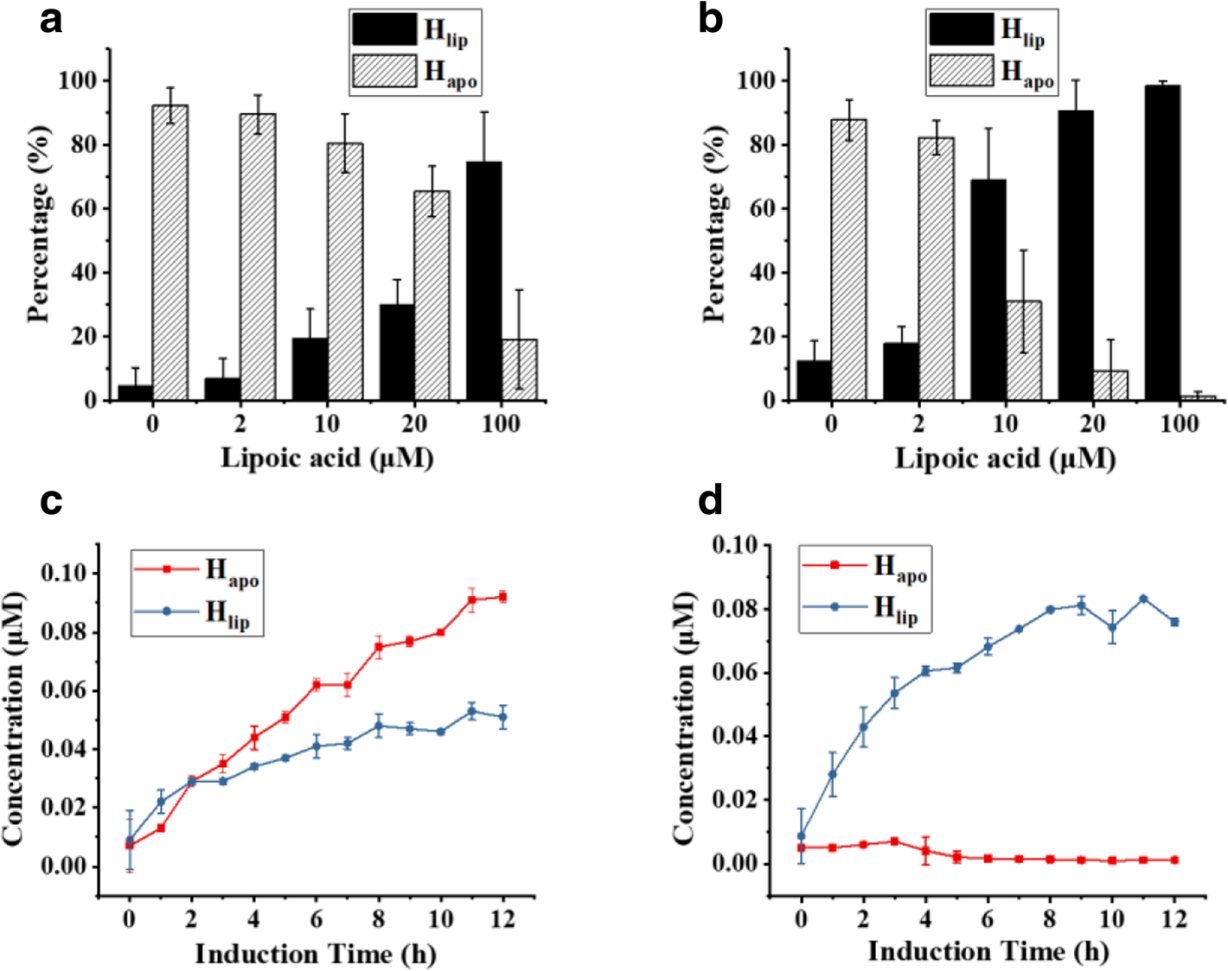
(**a**) Lipoylation ratio of H protein at different lipoic acid concentrations; (**b**) Lipoylation ratio of H protein with co-expression of LplA; (**c**) Time course for H protein without LplA co-expression; (**d**) Time course for H protein with LplA co-expression

The lipoylation ratio of H protein with different lipoic acid concentrations were tested using the new quantitative method. The concentration of lipoic acid did not have obvious effect on total concentration of H protein expressed (around 1.3 mg/mL), but strongly affected the lipoylation ratio (Fig. 5a). The lipoylation ratio of H protein was only 30% when 20 μM of lipoic acid was added in the culture, but increased to 80% with lipoic acid added up to 100 μM. In previous studies, different amounts of lipoic acid were added and this seems to be the main reason for the different results reported. Of course, the different strength of H protein expression may also affect the lipoylation ratio. 100 μM is a relatively high concentration. It is not known how the extracellular lipoic acid concentration will affect its intracellular concentration. Intracellular data would be of great interest.

### Co-expression of H protein and LplA increased the lipoylation and GCS activity

Considering the above results concerning H protein lipoylation it can be stated that a relatively high expression of H protein or an intracellular concentration of lipoic acid would be needed for a high GCS activity. The high expression of a single protein like the H protein may represent metabolic burden for the microbe. Alternatively, a high intracellular lipoic acid concentration is desirable and could be realized by adding a relatively high amount of exogenous lipoic acid. However, it will not only increase the costs of cultivation, but may course metabolic burden for the cells as well. A more favorable approach is to co-express the H protein with LplA at the same time. The lipoylation ratio of H protein with co-expression of LplA is shown in Fig. 5(b) for different lipoic acid concentration. By the co-expression of LplA, the lipoylation ratio was increased from 20 to 70% when 10 μM lipoic acid was added, and from 30 to 90% with 20 μM lipoic acid added, indicating significant improvements of lipoylation.

The time courses of H protein lipoylation with and without co-expression of LplA was followed in experiments with 20 μM lipoic acid added (Fig. 5c and d). Without co-expression of LplA, H_apo_ and H_lip_ were more or less the same in the first two hours after introduction with IPTG, but H_apo_ dominated for the rest time. Most of the H protein remained un-lipoylated and are thus not active. With the co-expression of LplA, the H protein was lipoylated quickly. H_lip_ was dominating in the whole process and nearly all the H protein was lipoylated after a few hours of expression of LplA.

**Fig. 6:**
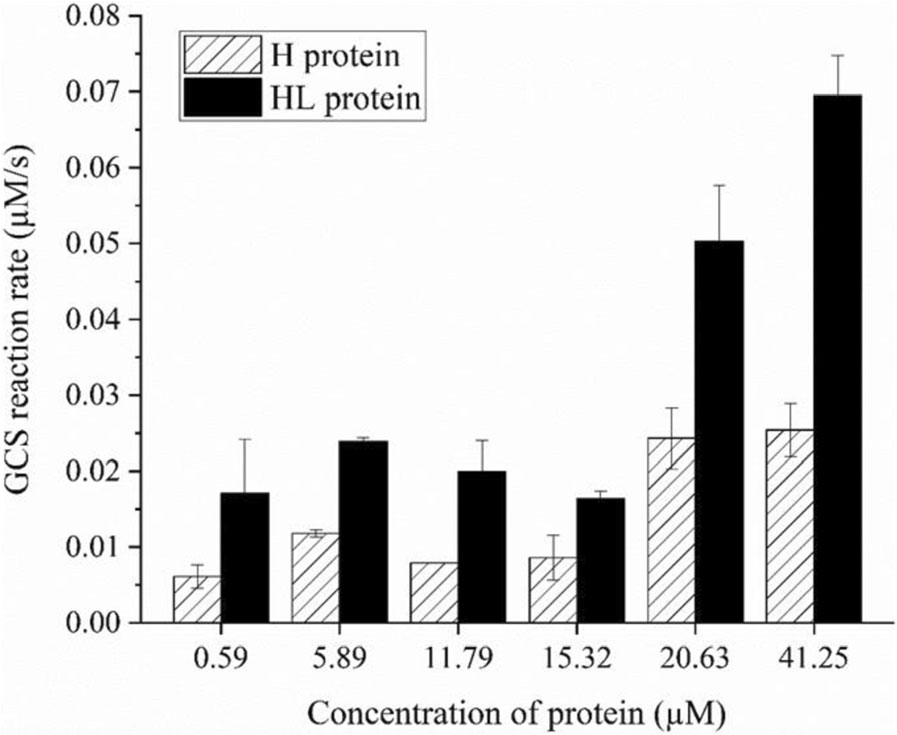
Glycine cleavage activity of GCS at different concentration of total H protein expressed with different strategies. HL protein was obtained by co-expression of H protein with LplA

The glycine cleavage activity of GCS with H protein expressed with different expression strategies was also measured with purified enzyme. Adding the same total amount of H protein, the activity of H protein co-expressed with LplA was 2–2.5 time higher that without LplA co-expression (Fig. 6). It should be mentioned that no lipoic acid was added during the activity assay. The differences observed in Fig. 6 result from the different lipoylation levels of H protein as shown in Fig. 5 with or without co-expression of LplA.

It is worth mention that H_apo_ has an inhibiting effect on the whole GCS activity as shown in Fig. 3. Without LplA co-expression H protein expressed in cultures with lipoic acid added at concentrations less than 100 μM mainly exists in the H_apo_ form which may seriously impair the GCS activity. Even with LplA co-expression the availability of lipoic acid seems also to be important for the lipoylation of H protein (Fig. 5b). It would be interesting to know the typical intracellular concentrations of lipoic acid, LplA and H protein in different cells or under different conditions to judge the relevance of the results reported in this work. It should be mentioned that to address different biological questions related to GCS a further differentiation and quantification of H_lip_ in its oxidized form (H_ox_), reduced form (H_red_) and intermediate form (H_int_) (Fig. 1) is also of great interest. Efforts are being made in our laboratory in this regards. With such a differentiation it would be possible to individually measure the enzyme activities of P, T, and L proteins and their joined effects on GCS. Potential inhibiting effects of H_apo_ protein on P, T and L proteins could be also studied one by one. With such information available, we can finally better understand the intracellular regulation of GCS and guide the design of synthetic formate utilization pathway with highly reverses activity of GCS.

## Conclusion

A new HPLC method has been developed for direct analysis and quantification of H_apo_ and H_lip_ from cell lysate without purification. This greatly facilitates the study of lipoylation of H protein in the GCS system. The lipoylation of H protein is inefficient at low temperature (e.g. 18 °C) and low extracellular concentration of lipoic acid (e.g. at less than 20 μM) under conditions studied in this work. We proposed the strategy of co-expressing LplA and significantly improved the lipoylation of H protein and GCS activity, even at low concentrations of lipoic acid. This work lays a quantitative foundation toward better understanding and reengineering the GCS system, e.g. for the use of formate and CO_2_ for biosynthesis.
